# Resistance Is Not Futile: The Role of Quorum Sensing Plasticity in *Pseudomonas aeruginosa* Infections and Its Link to Intrinsic Mechanisms of Antibiotic Resistance

**DOI:** 10.3390/microorganisms10061247

**Published:** 2022-06-18

**Authors:** Kayla A. Simanek, Jon E. Paczkowski

**Affiliations:** 1 Department of Biomedical Sciences, School of Public Health, University at Albany, Albany, NY 12201, USA; ksimanek@albany.edu; 2 Division of Genetics, Wadsworth Center, New York State Department of Health, Albany, NY 12208, USA

**Keywords:** antibiotic resistance, virulence, quorum sensing

## Abstract

Bacteria use a cell-cell communication process called quorum sensing (QS) to orchestrate collective behaviors. QS relies on the group-wide detection of extracellular signal molecules called autoinducers (AI). Quorum sensing is required for virulence and biofilm formation in the human pathogen *Pseudomonas aeruginosa*. In *P. aeruginosa*, LasR and RhlR are homologous LuxR-type soluble transcription factor receptors that bind their cognate AIs and activate the expression of genes encoding functions required for virulence and biofilm formation. While some bacterial signal transduction pathways follow a linear circuit, as phosphoryl groups are passed from one carrier protein to another ultimately resulting in up- or down-regulation of target genes, the QS system in *P. aeruginosa* is a dense network of receptors and regulators with interconnecting regulatory systems and outputs. Once activated, it is not understood how LasR and RhlR establish their signaling hierarchy, nor is it clear how these pathway connections are regulated, resulting in chronic infection. Here, we reviewed the mechanisms of QS progression as it relates to bacterial pathogenesis and antimicrobial resistance and tolerance.

## 1. Introduction

According to the Centers for Disease Control and Prevention, *Enterococcus faecium*, *Staphylococcus aureus*, *Klebsiella pneumoniae*, *Acinetobacter baumannii*, *Pseudomonas aeruginosa*, and *Enterobacter* spp. (ESKAPE) represent a significant threat to human health because they are commonly found to be resistant to many of the clinically relevant antimicrobial therapies—either bactericidal or bacteriostatic—currently used to treat infections (Table 1) [1,2]. *P. aeruginosa* is a Gram-negative bacterium that is ubiquitous in the environment. The soil is the natural reservoir for *P. aeruginosa*, but it has the remarkable capacity to grow in nutrient-limited environments, such as the reservoirs of water around sinks and in hospital ventilators. As a result, it has become one of the most common nosocomial (hospital-acquired) pathogens and is the primary cause of ventilator-associated pneumonia (VAP) in the United States. Additionally, *P. aeruginosa* represents a significant threat to hospitalized patients suffering from burn wounds, as well as those with implanted medical devices, such as catheters.

The persistence of *P. aeruginosa* as an opportunistic pathogen is most readily observed in the immune-compromised, in patients with cystic fibrosis (CF), and in those with underlying pulmonary disorders [3]. Once established in the human lung, *P*. *aeruginosa* is difficult to eliminate, which is directly related to the intrinsic resistance mechanisms used by *P*. *aeruginosa* to evade treatment. These mechanisms include but are not limited to, low permeability of the cell membrane and multiple efflux pump systems [4,5,6]. Moreover, *P*. *aeruginosa* forms robust biofilms, which are multicellular communities that make bacteria more recalcitrant to bactericidal treatments and, most critically, are important for establishing a long-term, chronic infection in the host lung [7,8,9,10]. Indeed, treatment outcomes for *P. aeruginosa* infections are varied because approximately 30% of all clinical isolates are resistant to a single antibiotic, with 15% of clinical isolates being multi-drug resistant [11]. In this review, we analyze and discuss the literature detailing the molecular basis for virulence progression in *P. aeruginosa* in different, clinically relevant settings as it relates to quorum sensing (QS) and antibiotic resistance.

## 2. Clinical Ubiquity and Transmission

### 2.1. P. aeruginosa and Pulmonary Infections

Approximately 15% of all adults in the United States suffer from an underlying pulmonary condition that affects their ability to clear infections [12]. In addition to other ESKAPE pathogens, *P. aeruginosa* is one of the most common causes of hospital-acquired lung infections and VAP in the United States, resulting in approximately 32,000 new infections per year with mortality rates ranging between 15–30%, typically the result of bacteremia, shock, and/or hypoxia [12,13]. These infections account for approximately 20% of all nosocomial pneumonia cases [14]. Consequently, *P. aeruginosa* infections in a hospital setting present a large financial burden on the healthcare system. On average, this results in a $40,000 increase in the cost of hospital stays per patient [15].

*P. aeruginosa* can cause two different types of infections that manifest differently in the host lung: (i) short-term colonization with underlying disease exacerbation, often referred to as an acute infection, followed by a clearing of the infection or (ii) long-term, chronic persistence in the lung, typically associated with the presence of biofilms (high levels of the extracellular polymeric substance (EPS) alginate gives the characteristic mucoid biofilm morphology in these isolates) and the employment of antibiotic resistance mechanisms [12]. Most hospital-acquired lung infections fall into the former category, as the infections can be cleared within one month of initial infection, typically with antibiotic treatment. However, long-term, chronic infections can persist for months to years, with different treatment regimens resulting in different clinical outcomes. Indeed, the genetic variability and the environmental adaptability of *P. aeruginosa* make treatment difficult as different isolates have evolved in a variety of niches to develop resistance to a range of treatments. Developing treatment options that target a mechanism of virulence or pathogenesis progression that is required across different environmental niches is critical to addressing the urgent need for new therapeutics to treat *P. aeruginosa* infections. Additionally, should these targeted behaviors be dispensable for growth, the rapid proliferation of resistance mechanisms against these therapeutics should not be observed, effectively mitigating the risks associated with current bacteriostatic or bactericidal treatments. Prevention and early treatment are the best defenses to minimizing the risk of mortality due to hospital-acquired *P. aeruginosa* infections.

Long-term, chronic infections are most readily observed in patients suffering from chronic obstructive pulmonary disorder (COPD) and CF—two of the most well-studied pulmonary disorders associated with *P. aeruginosa* infections [12]. While COPD is a pulmonary condition associated with environmental factors such as smoking and prolonged exposure to air irritants, it bears some similar clinical manifestations to the genetic disorder giving rise to CF. COPD is marked by a progressive cough and mucus production that worsens over time, leading to exacerbation of the disorder and, ultimately, requiring frequent hospital visits for treatment intervention to ameliorate symptoms. These visits, in addition to other environmental factors, are often where *P. aeruginosa* infections are acquired and, as the disease progresses with repeated hospitalizations, the infections become more difficult to clear. A longitudinal study of hospitalized patients suffering from COPD revealed that 31% of patients suffered from a *P. aeruginosa* infection and, within that subset of patients, there were multiple reoccurrences of infection by different strains of *P. aeruginosa* [16]. These re-infections led to a marked increase in the risk of COPD exacerbation, which, given the cyclical nature of the disease, resulted in the patients becoming more susceptible to future infections. Furthermore, it was found that multiple re-infections increased the risk of acquiring multi-drug resistant strains, which is associated with a 6-fold higher mortality risk—a staggering statistic, considering that more than 60 million Americans suffer from COPD [13]. Researchers and clinicians have been unable to gain traction on a viable treatment plan because of strain heterogeneity at the genetic level and a variety of host factors, which are addressed below.

While relatively little is known about *P. aeruginosa* infection progression in individuals suffering from COPD due to the many confounding factors that exist around the disease related to the host, more is known about the molecular basis of the progression of *P. aeruginosa* pathogenesis in patients suffering from CF. CF is an autosomal recessive disorder that occurs in 1 in 2500 births and is the result of at least one or more of 1500 possible mutations in the *CFTR* (cystic fibrosis transmembrane conductance regulator) gene [17]. CF is characterized by a decrease in the volume of periciliary fluid in the lower respiratory tract, which leads to an inability to expel pathogens through mucociliary clearance. In turn, much like in COPD patients, the thick mucus layer that develops because of the defect in ciliary clearance allows for the colonization of the airway by *P. aeruginosa*. Pathogen colonization can occur within the first year of life for a CF patient. Despite early intervention efforts that have delayed the initial onset of infection, *P. aeruginosa* remains the most common pathogen found in CF patients. Over time, as *P. aeruginosa* transitions from acute, intermittent infections to long-term, chronic infections, it becomes the dominant species in the lung for 80% of CF patients, with a large percentage of isolates that are multi-drug resistant. A study conducted between 2009 and 2020 collected 721 CF *P. aeruginosa* isolates and determine their susceptibility to different antibiotics. Remarkably, 496 of the 721 isolates were resistant to the three major antibiotic classes (fluoroquinolones, β-lactams, and aminoglycosides) [11]. These results demonstrated that resistance to fluoroquinolones has trended upward in the past decade, while susceptibility to aminoglycosides remained relatively steady. Additionally, based on CDC research and surveillance, resistance to β-lactams is becoming prevalent in hospitals due to horizontal gene transfer of plasmid-borne β-lactamases [2].

Despite medical intervention, CF patients often have persistent, chronic infections by early adulthood. It is often the case that patients are re-colonized by a different strain of *P. aeruginosa* after an acute, intermittent infection. However, re-colonization can also occur via the identical strain from the initial infection, typically the result of a constant environmental source or a reservoir within the host that was not effectively eradicated by the immune system or treatment. It has been suggested that the paranasal cavity of a patient could be one such niche where *P. aeruginosa* can evade treatment, allowing for subsequent re-infection [18,19]. However, the ability for an infection to be the result of a mixed-strain population leads to a higher treatment burden than patients with a single-strain infection, indicating a need to implement drastic infection control measures in the clinic [20]. Indeed, for many years it was believed that patient-to-patient strain sharing was minimal, but a retrospective study relying on whole-genome sequencing of CF pulmonary isolates from children in Toronto, Canada found that 41% of isolates were shared between patients, indicating an unprecedented level of patient-to-patient isolate transmission [21]. Irrespective of the source, the effects of chronic infections remain deleterious to the patient, resulting in inflammation of the lung tissue, lung necrosis, and hypoxia. Ultimately, these patients succumb to the long-term burden of *P. aeruginosa* infections.

In Section 3.6, the role of *P. aeruginosa* strain heterogeneity as it relates to lung infections will be further explored. Discussion will also focus on the transition from acute, intermittent infection to chronic, long-term infections, the role of QS and biofilm formation in this process as well as other intrinsic mechanisms of antibiotic resistance that complicate treatment.

### 2.2. P. aeruginosa Is More Than Just a Lung Pathogen

The pervasiveness of *P. aeruginosa* infections extends beyond the pulmonary system. *P. aeruginosa* is also adept at colonizing the wounds of burn victims and the urinary tracts of patients with implanted catheters [22,23,24,25]. As expected, the environmental niche at the site of infection is different for all the cases highlighted in this review, requiring a fundamental understanding of the effects of the environment on virulence signaling [26]. However, there is one common thread linking different *P. aeruginosa* infections: biofilm formation is the primary driver of pathogenicity in patients, albeit by different mechanisms. In this section, the risks associated with *P. aeruginosa* infection in burn wounds and urinary tract infections (UTIs) will be discussed [24,27,28]. In Section 3.7, we will analyze the role of QS signaling in these types of infections.

#### 2.2.1. Burn Wounds

Infections at the site of burn wounds, especially in patients suffering extensive damage to more than 40% of their body, are one of the most common causes of sepsis and mortality in burn victims [29]. Additionally, infection at the site of skin grafts is one of the leading causes of graft failure, presenting a substantial cost and treatment burden on the healthcare community. The two most common pathogens found at the initial site of infection are *S. aureus* and *P. aeruginosa*. *S. aureus* is thought to be the initial colonizer of burn wounds because it is a natural constituent of skin microflora [30]. However, secondary colonization by *P. aeruginosa* can occur in as little as seven days, post-injury, leading to a more invasive, chronic infection and, in extreme cases, sepsis [22]. It is at this point that *P. aeruginosa* represents the primary microorganism in the wound and is the main driver of bacteremia.

Approximately 2.5 million people in the United States suffer from a burn wound that requires medical intervention, with nearly 10% of patients requiring hospitalization [29]. The previous three decades have seen the implementation of many early intervention mitigation efforts to prevent infections in trauma units, but the trends remain the same: *P. aeruginosa* is one of the primary causes of infections in burn victims [31,32]. Multiple factors play a role in the risk of infection for a patient suffering from burn trauma. These include the total surface area of the burn and the length of hospitalization. Simply, more exposed wounds in conjunction with a prolonged stay in an environment where multi-drug resistant bacteria are common is a significant threat to the patient [33]. As with the immunocompromised and patients with underlying pulmonary disorders, burn victims often acquire *P. aeruginosa* from the local hospital environment. Contaminated bedding materials, infected cohabitating patients, or any number of required medical practices that might expose the patient to a pathogen are the main factors that drive nosocomial infection rates [34]. Extreme isolation methods are often required to reduce the potential for bacterial infection. However, should such mitigation efforts be delayed in implementation or prove not to be effective, an intensive course of antibiotic treatment is required.

A variety of burn models have been developed to study infection progression. These studies have shown that, like other disease models, *P. aeruginosa* can rapidly colonize and become the predominant species at the site of infection after its introduction and this is dependent on the overexpression of genes associated with virulence and biofilm formation [27,35,36]. In Section 3.5.3 and Section 4.1, the molecular basis for biofilm formation and its role as an intrinsic driver of antibiotic resistance, respectively, will be discussed.

#### 2.2.2. UTIs

Catheter implantation is common among hospitalized individuals, with up to 25% of patients having undergone the procedure. In these cases, implantation lasts upwards of four days [37]. Catheterization can also be long-term, as individuals living in nursing homes and assisted-living environments can have implanted catheters for months to years, depending on their condition. Catheter-associated UTI is one of the most common nosocomial infections, representing 40% of all such infections [38]. *Escherichia coli* and *Enterococci* spp. are the main pathogens in this environment [37]. However, *P. aeruginosa* is responsible for 12% of all nosocomial UTIs [39]. Together, these infections are responsible for dramatically increasing the length of hospital stays for already compromised individuals, causing or contributing to approximately 7500 deaths per year [38].

Catheter implantation, much like intubation while on a ventilator, creates an environment in the host that is amenable to pathogen colonization, proliferation, and subsequent biofilm formation. A survey of clinical isolates of *P. aeruginosa* obtained from patients with UTIs revealed broad resistance to cefotaxime, ampicillin, tetracycline, tobramycin, amikacin, meropenem, and imipenem [40,41]. Subsequent testing of these isolates under conditions with low nutrient availability and reduced oxygen concentrations like that of the urinary tract, as well as conditions that led to the transition to the biofilm state, increased the resistance of these strains to the above antibiotics by nearly 6000-fold. Furthermore, in vivo and in vitro models showed that laboratory strains of *P. aeruginosa* formed biofilms in the absence of EPS production (Pel, Psl, and alginate—further discussed below) required for biofilm formation under standard laboratory conditions. Interestingly, the EPS components of a biofilm are required for colonization in other clinically relevant models. Subsequently, it was shown that biofilm formation can be induced by murine and human urine in strains of *P. aeruginosa* that do not produce EPS, with the likely driver of the phenotype being extracellular DNA (eDNA) from lysed cells [24,39]. These results indicated that *P. aeruginosa* biofilm formation, while important in the context of nearly all infections, can proceed via different mechanisms depending on the local environment. This is highlighted in this review to illustrate the remarkable adaptability and plasticity of the signaling networks that drive pathogenicity in *P. aeruginosa*. It is necessary to understand these pathways and how they might be altered under different conditions to find a commonality to help mitigate the increasing issue of antibiotic resistance in nosocomial infections.

## 3. Pathogenicity and Virulence

### 3.1. Quorum Sensing (QS): For the Many, Not the Few

QS is a process of bacterial cell-cell communication that controls virulence and biofilm formation in many bacterial species, including *P. aeruginosa*. QS relies on the production, accumulation, detection, and population-wide response to extracellular signal molecules called autoinducers (AI). QS allows bacteria to synchronously alter gene expression patterns that underpin collective behaviors including, but not limited to biofilm formation, virulence factor production, and pathogenesis. QS is responsible for releasing public goods that are beneficial to kin, and potentially non-kin, and as a result, it plays an important role in shaping microbial community architecture.

### 3.2. The Complex Regulatory Network of QS in P. aeruginosa: The Key Determinants to QS Progression

**Figure 1:**
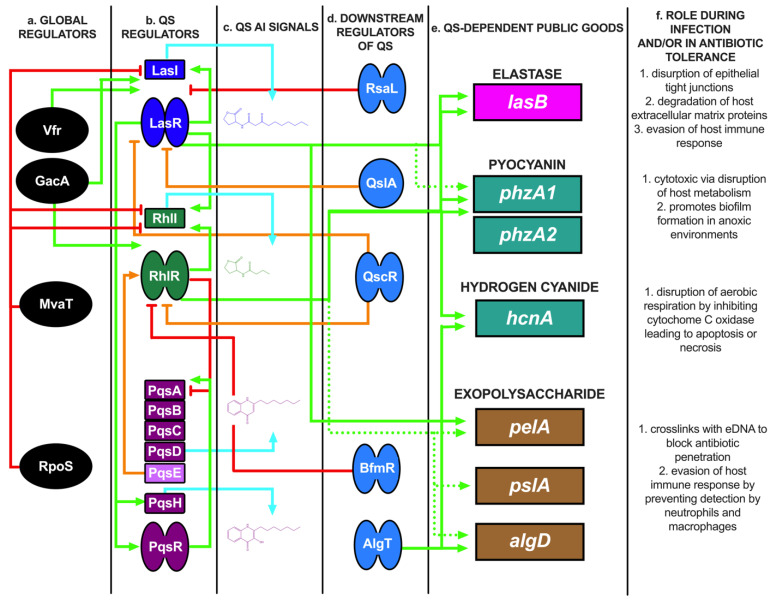
What regulates the regulators? Six steps from global gene regulation to infection and antibiotic tolerance. (**a**) A selection of well-characterized global transcriptional regulators that directly regulate the expression of the *las* and *rhl* QS systems. (**b**) The three main branches of QS regulators all regulate their own signal production through an autofeedback mechanism that results in the upregulation of their respective AI signals, as shown in (**c**). There is an extensive interplay between the three QS pathways. PqsE is colored light purple, distinct from the rest of the PQS pathway, because of its role outside of the biosynthesis of PQS related to regulating RhlR function. (**c**) The three main AI signals that bind to their cognate receptors in (**b**). HHQ, the precursor to PQS synthesis is shown because it can also act as an activator of PqsR. (**d**) Downstream regulators may or may not be QS regulated, but they exert their influence on QS by repressing the activation of QS through transcriptional regulation or binding to the receptors to disrupt their activation. (**e**) The totality of the regulation shown in a-d manifests itself in the timely expression of QS-dependent public goods. For simplicity, we highlight only the genes discussed in this review. (**f**) A summary of the effects each of the QS-dependent public goods has during host infection. Red bars indicate transcriptional repression. Green arrows indicate transcriptional activation. Blue arrows indicate AI production and binding. Orange bars and arrows indicate protein-protein interactions that result in activation and repression, respectively. Dotted green lines indicate indirect transcriptional activation.

LasR and RhlR detect N-(3-oxododecanoyl)-L-homoserine lactone (3OC_12_HSL) and N-butyryl-L-homoserine lactone (C_4_HSL), respectively. 3OC_12_HSL and C_4_HSL are produced by the synthases LasI and RhlI, respectively (Figure 1) [42,43,44,45,46,47]. LasR and RhlR belong to a group of soluble transcription factor receptors known as LuxR-type receptors, which have a variable ligand-binding domain (LBD) and a well-conserved helix-turn-helix DNA binding domain (DBD) [48]. Upon signal recognition, LasR and RhlR activate hundreds of genes, many of which are involved in pathogenesis and biofilm formation [46,49,50,51,52,53]. While some signal transduction pathways follow a linear circuit as phosphoryl groups are passed from one carrier protein to another, ultimately resulting in upregulation or downregulation of target genes, the QS system in *P*. *aeruginosa* is best described as a dense network of receptors and regulators with interconnecting regulatory systems [54,55,56,57,58,59,60,61,62] (Figure 1a–d). Indeed, in addition to LasR and RhlR, there is a third QS system regulated by the signaling molecule PQS (2-heptyl-3-hydroxy-4-quinolone) (Figure 1d) [63,64,65,66,67]. LasR and RhlR positively and negatively regulate receptor and signal production in the PQS pathway, respectively [63,68]. The biosynthetic gene operon consisting of *pqsABCDE*, in addition to *pqsH*, is responsible for the production of the PQS. PqsR is the receptor for the PQS molecule. In addition to activation by PQS, the PQS precursor molecule, HHQ (4-hydroxy-2-heptylquinolone), activates PqsR, albeit with reduced affinity [63,65]. PqsR is a member of the LysR family of transcription factors and regulates genes involved in iron acquisition and secretion. Additionally, activated PqsR plays an important role in the upregulation of its own AI biosynthetic pathway. This is a hallmark of QS signaling: an activated receptor upregulates the transcription of the synthase(s) responsible for the biosynthesis of the AI, creating a positive feedback system [54]. This requires multiple external regulators to exert their influence on the system to prevent uncontrolled QS [67]. In addition to the core QS network, global regulators such as Vfr, GacA, RpoS, MvaT, and BfmS/R as well as QS-controlled regulators RsaL, QscR, and QslA, also control the expression of the receptors in a cell-density-dependent manner (Figure 1) [64,69,70,71,72,73,74,75].

### 3.3. The Rhl System Can Bypass the Requirement for Activation by the Las System

Recent discoveries suggest that the canonical pathway of RhlR activation via LasR might not be as straightforward as previously thought; research on clinical isolates of *P. aeruginosa* has shown that RhlR-dependent transcription is upregulated in the presence of *lasR* inactivating mutations, indicating that RhlR can function in the absence of its proposed activator, LasR [18,76,77,78,79,80]. Furthermore, nutrient limiting conditions mimic those that *P. aeruginosa* would face in a host when establishing an infection (Box 1). Specifically, under phosphate-limiting conditions, the *rhl* system can function in the absence of the *las* system, indicating that there are conditions in which RhlR can drive virulence factor production [81]. Additionally, synthase-receptor pairs typically have overlapping phenotypes in bacterial QS signaling, but in the case of the *rhl* system, RhlR-directed virulence factor production has been shown to be independent of its partner synthase, RhlI [53,82,83,84].

Box 1 **Limitations in our understanding of QS in different environments and the barriers to the discovery of effective treatments. Outstanding questions in the field of QS signaling.**

- *P. aeruginosa* is found in several different environments, both inside and outside the host. Due to its large genome, it is highly adaptable to thrive under different conditions and stresses. Is there a standard growth condition for laboratory experiments that represents a viable way to assess virulence factor production that is more clinically relevant?
- A variety of host infection models exist for most of the clinically relevant infections caused by *P. aeruginosa*. How closely do these resemble the human host? What are the host factors that complicate understanding *P. aeruginosa* pathogenesis?
- The efficacy of anti-QS therapies has been assessed in vitro with modest effects but has failed at limiting virulence in vivo. What are the gaps in our understanding of the system that result in this disconnect? Have our laboratory strains been tailored to meet our scientific research questions but are no longer capable of addressing our clinical needs?

### 3.4. QS-Driven Pathogenesis Proceeds through RhlR and PqsE in a PQS-Independent Manner

Above, *pqsE* was described as part of the *pqs* operon, which is responsible for the production of the signaling molecule, PQS. However, the role of PqsE in this pathway, and QS in general, has been elusive until recently [83,84]. PqsE is a metallo-β-hydrolase and the proposed role of PqsE in PQS production is via the conversion of 2-aminobenzoylacetyl-CoA (2-ABA-CoA) to 2-aminobenzoyl acetate (2-ABA) [85]. This reaction has been confirmed in vitro. However, a Δ*pqsE* mutant or a strain expressing a catalytically inactive variant of PqsE makes wild-type (WT) levels of PQS, presumably due to the presence of other thioesterase(s) that can perform the same function of converting 2-ABA-CoA to 2-ABA [85]. Consistent with this, a Δ*pqsE* mutant cannot be complemented by the addition of PQS; the addition of the signaling molecule cannot recover the loss of pyocyanin production, which is an important virulence factor (see Section 3.5.2, in this background). The loss of virulence factor production in a Δ*pqsE* mutant is consistent with the avirulent, non-pathogenic phenotype observed in a murine infection model when mice were challenged with the mutant strain [82]. These findings indicate that PqsE plays a role in virulence factor production and pathogenesis outside of its proposed role in the PQS biosynthetic pathway, where it was previously shown that PqsE regulates RhlR function [86]. Ultimately, the mechanism of PqsE-dependent regulation of RhlR was shown to be through binding to RhlR, with PqsE-RhlR complex formation altering the affinity of RhlR for target promoter DNA (Figure 1b) [83]. In so doing, PqsE and RhlR co-regulate genes involved in virulence factor production and pathogenesis. Interestingly, RhlR-dependent regulation can proceed through at least two distinct mechanisms at different promoters. (i) RhlR can function without PqsE bound, presumably as a homodimer like other LuxR-type receptors, since a more dramatic decrease in RhlR-dependent signaling in a *rhlR* mutant than a *pqsE* mutant was observed. This indicated that RhlR can function as a transcription factor in the absence of PqsE, albeit in a reduced capacity. (ii) RhlR can function without the RhlI-synthesized C_4_HSL, as *rhlI* was dispensable for the regulation of *hcnA* as seen in RNA-seq data (Section 3.5.4). Based on this information, we proposed that the final, most important step in the progression of QS under most conditions is the interaction of PqsE with RhlR, leading to the expression of factors required for the transition to chronic infections in the host. Understanding the signaling systems required to function under these conditions will be important in establishing QS as a potential target for developing new anti-infectives [83,84].

### 3.5. Disease Relevant Virulence Factors

At high cell density, *P. aeruginosa* produces several public goods that are beneficial only when produced as a collective, allowing them to engage in community behaviors that will ensure their success in different environments (Figure 1e). This is contrary to many other well-known pathogens that rely on only one or a few virulence factors to cause disease, and is likely the consequence of the relatively large genome of *P. aeruginosa*. In this regard, *P. aeruginosa* secretes many factors that are involved in biofilm formation (Psl, Pel, and alginate), cytotoxicity (elastase, hydrogen cyanide, alkaline proteases, and pyocyanin), surfactant production (rhamnolipids), and micronutrient, such as iron, scavenging (pyoverdine), among others (see [87] for a thorough review of other important *P. aeruginosa* virulence factors). Each of these factors plays an important role at different stages of infection. Collectively, these secreted factors perform at least one or more of the following roles: (i) target host tissue for damage, (ii) evade host immune defenses, and (iii) establish or maintain the infection. For the scope of this review, we will highlight a few QS-dependent factors and the role an adaptable QS network plays in their timely expression during infection.

#### 3.5.1. Elastase

Elastase is perhaps one of the most well-studied *P. aeruginosa* virulence factors. The regulation of elastase by the *las* QS system is how this arm of the QS pathway received its name. However, it is worth noting that elastase secretion can still occur in a *lasR* mutant and that only when both *lasR* and *rhlR* are absent is elastase production abrogated [43]. Furthermore, the interaction between PqsE and RhlR is required for optimal expression of *lasB* [83].

Elastase is a metallo-protease that uses divalent cations, specifically Zn^2+^ and Ca^2+^, to carry out its function in degrading important components of the extracellular matrix, such as elastin, collagen, laminin, and fibronectin of the host cells, ultimately leading to tissue damage [88]. Additionally, *P. aeruginosa* elastase can disrupt the tight junctions in human nasal epithelial cells, thereby disrupting the signal transduction pathways that regulate the epithelial barrier [89]. An early study of important virulence factors controlled by QS showed that deletion of *lasB* led to a reduction in the bacterial load during initial respiratory tract infections in murine infection models, indicating a primary role in establishing infections via tissue damage [90]. However, *P. aeruginosa* evasion of host defenses might also be mediated by LasB. LasB can degrade components of both the innate and adaptive immune system, targeting the cytokines and chemokines TNF-a, IFN-y, IL-2, and IL-8 [91,92]. In addition to targeting host factors, LasB can also modulate the recognition of *P. aeruginosa* by host defenses. The flagellum, an important driver of bacterial motility, is recognized as a foreign antigen by toll-like receptor 5 (TLR5), inducing a proinflammatory response by the host. LasB can degrade flagellin in vitro and alter the host response within the context of flagellin-mediated host detection [93]. It was shown that incubating purified LasB with the flagellin resulted in the rapid degradation of the flagellin and this resulted in a concomitant decrease in the immune response via the detection of IL-8 in A549 human airway epithelial cells [93]. Thus, *P. aeruginosa* can escape flagellin-dependent immune recognition by decreasing the concentration of free flagellin that can act as an inducer of immunogenicity.

The expression and role of LasB in non-mucoid and mucoid clinical isolates have been a source of mystery in the field [3,94]. The mechanism by which strains promote the mucoid phenotype via alginate production in *P. aeruginosa* and, in turn, reduce the secretion of virulence factors such as elastase is unclear. As noted above, LasB has multiple roles in different stages of infection, making it difficult to speculate about its role in any one clinical isolate from a given source. However, the relationship between high elastase-producing clinical isolates and an increase in the risk of mortality associated with the infection has been established. For example, a prospective study of 238 clinical isolates from ICU patients in tertiary-care centers revealed that 75% of the isolates retained their ability to produce elastase and, based on whole-genome sequencing, this correlated with having an intact *las* system [95]. These isolates were associated with an increase in 30-day mortality in the patients screened. Furthermore, murine models with high elastase producers revealed a higher bacterial burden than low elastase producers, mimicking what was observed in laboratory strains. A similar correlation was observed in clinical isolates obtained from patients suffering from chronic rhinosinusitis (CRS) [19,89,96]. This condition is of great importance because these infections often coincide with the ability of *P. aeruginosa* to evade host detection or antibiotic treatment and allow for adaptation that promotes the transition from acute and intermittent to chronic lung infections in patients with CF and COPD. *P. aeruginosa* elastase secretion causes CRS by compromising the mucosal barrier; a study of 21 patients suffering from CRS showed a strong correlation between in vitro elastase activity and mucosal barrier disruption, thus, linking disease severity with elastase activity [19].

#### 3.5.2. Pyocyanin

Pyocyanin is one of at least five phenazine molecules produced by *P. aeruginosa*. Phenazines are heterocyclic, nitrogen-containing secondary metabolites. Pyocyanin—the product of a complex biosynthetic process starting with chorismic acid—is a redox reactive molecule that, when in the oxidized state, gives *P. aeruginosa* cultures their characteristic blue-green hue. The biosynthesis of pyocyanin is complex. Phenazine production is dependent on two duplicate operons, consisting of *phzA1-G1* and *phzA2-G2*, as well as *phzH, phzM,* and *phzS.* Furthermore, the QS regulation that underpins the expression of these genes is sophisticated, with all of the major QS regulators implicated in their expression [86,97,98,99]. However, it was recently demonstrated that simply co-expressing RhlR and PqsE in an otherwise QS-deficient background of *P. aeruginosa* could drive pyocyanin production [83]. Pyocyanin production was dependent on the physical interaction between RhlR and PqsE; when a PqsE variant that could no longer interact with RhlR in vitro was introduced on the chromosome of *P. aeruginosa*, pyocyanin was no longer produced. This indicated that the PqsE-RhlR interaction is sufficient for pyocyanin production. Thus, it is likely that the other QS regulators exert their influence on pyocyanin production via the regulation of RhlR and/or PqsE. However, little is known about the complex regulation of both operons, which share 98% similarity but have divergent promoters [52]. Curiously, under phosphate-limiting conditions, QS-dependent regulation of pyocyanin production appears restricted to the *phzA1* and not *phzA2* operon [77,81]. The regulation of the two operons and how they evolved are the focus of ongoing investigations.

The requirement of pyocyanin for full virulence in various animal models is unambiguous and thoroughly reviewed [40]. The cytotoxic effects of pyocyanin on host cells rely on the production of reactive oxygen species (ROS), which can significantly alter cellular metabolism [100]. The toxic effects of pyocyanin on pulmonary epithelial cells and neutrophils have been established, indicating a significant role for pyocyanin in the pathophysiology of disease progression. Indeed, pyocyanin levels typically correlate with disease severity in the lung; high levels of pyocyanin correlate with periods of pulmonary exacerbation in patients with CF [101]. High levels of pyocyanin induce mucin production, which can be detrimental to these patients, as uncontrolled mucus production can restrict airways and promote additional attachment [17,102]. Pyocyanin has multiple negative effects on respiratory epithelial cells via ROS and the reduction of NAD(P)H in host cells, resulting in (i) inhibition of catalase, resulting in increased levels of hydrogen peroxide in the cell, (ii) inhibition of ciliary beat frequency, resulting in the failure to clear *P. aeruginosa* from the airway, and (iii) inhibition of the ⍺1 protease inhibitor, resulting in enhanced elastase-mediated tissue damage [103,104]. Furthermore, pyocyanin was shown to suppress the acute inflammatory response by driving neutrophil apoptosis and reducing localized lung inflammation, which further assists *P. aeruginosa* in evading the immune response [105].

For several years, it was believed that pyocyanin was simply a secondary metabolite that did not serve a basic function for *P. aeruginosa* and that its characteristics as a cytotoxic factor were happenstance. The complex regulation of phenazines indicates that this is likely not the case and recent research indicates that pyocyanin plays an important role in many physiological traits as well as ensuring the survival of *P. aeruginosa* in different environments, particularly the host. Related to its ability to facilitate extracellular electron transfer (EET), pyocyanin was shown to play a critical role in mediating biofilm development by promoting survival in anoxic environments [104]. Because of its effects on metabolism, *P. aeruginosa* must balance the extracellular levels of pyocyanin; under different conditions, pyocyanin can be toxic to the cells producing it or it can promote viability and biofilm growth. Indeed, pyocyanin production was shown to stimulate cell lysis, which led to the expulsion of DNA into the extracellular matrix (eDNA). eDNA is necessary for biofilm formation [106]. Thus, it was hypothesized that early cell death during biofilm development played a crucial role in sustaining metabolism to support further biofilm development. Subsequent research into the role of pyocyanin in biofilm formation revealed that pyocyanin interacted with eDNA to enhance its role as an electron shuttle [104]. Ultimately, the balance between the toxic and beneficial traits conferred by pyocyanin is critical to the survival of *P. aeruginosa* in the host [103]. The role of phenazines in biofilms and how it relates to antibiotic resistance is discussed in Section 4.1.

#### 3.5.3. Alginate, Psl, and Pel

While pyocyanin and elastase likely play critical roles in all stages of infection, their expression in clinical isolates varies to different degrees. It is likely that they are required for the initial infection and colonization and then, depending on the host environment, signaling will adapt to meet the needs of *P. aeruginosa*. However, biofilm formation is vital to the development of chronic infections. One of the most common mutations that leads to the mucoid state of *P. aeruginosa* is the inactivation of the MucA anti-sigma factor, which regulates AlgT, the master regulator of alginate biosynthesis [107]. In the absence of functional MucA, AlgT is hyperactive and promotes the transcription of not only itself, but also the entire 12-gene alginate regulon (*algD*, *alg8*, *alg44*, *algK, algE, algG, algX, algI, algJ, algF, algA,* and *algC)* [16,108,109]. Curiously, ectopic overexpression of *algT* was lethal to *P. aeruginosa* in a clinical strain background lacking functional MucA, indicating that other factors exist that help maintain *algT* levels, even in a *mucA* deficient background [110].

Despite the consistent upregulation of alginate in strains of *P. aeruginosa* from chronic infections, it appears to play a more important role in biofilm maturation and expansion. Conversely, Pel (the biosynthetic operon *pelA-G*) and Psl (the biosynthetic operon *pslA-O*) play a more important role in biofilm initiation. All three are exopolysaccharides: alginate is an O-acetylated 1–4 linked D-mannuronic acid, Pel consists of 1–4 glycosidic linkages of *N*-acetylglucosamine and *N*-acetylglucosamine, and Psl consists of repeating pentasaccharides of D-mannose, D-glucose, and L-rhamnose. Pel and Psl are key to early-stage biofilm development, via surface adhesion and maintaining cell-cell contacts in the biofilm [8,111,112]. The deletion of the *psl* operon in mucoid isolate strains that overexpress alginate leads to a dramatic decrease in overall biofilm formation [113]. While their expression is strain-dependent, Pel and Psl each play a critical role in the clinical manifestation of biofilms. Both Psl and Pel are important for resisting treatment with antibiotics and conferring fitness in a biofilm [7,41,114,115]. A recent study showed that flagellar mutants overexpressed Pel and Psl in a surface contact-dependent manner and that these flagellar mutants were selected at a high frequency in biofilms, indicating that Pel and Psl provided a fitness benefit in the sessile lifestyle of *P. aeruginosa* [111]. Indeed, it was discovered that Psl can sequester iron, a vital micronutrient that is limited in the environment, which, in turn, can stimulate further Psl-dependent biofilm formation [116]. In total, *P. aeruginosa* forms robust biofilms due to the presence and the differing roles of the EPS.

Biofilm formation and development are controlled by several factors in addition to alginate, Psl, and Pel, including protein components such as LecAB and CdrA, eDNA, the small molecule c-di-GMP, and rhamnolipids [113,117,118,119]. Interestingly, despite the variation in the requirements of different EPS constituents in a wide range of clinical isolates, the other components of the biofilm appear to be just as critical in biofilm development across isolates. For example, CdrA is a novel protein that does not possess any sugar-binding motifs or lectin binding domains but still possesses the ability to crosslink Psl. Clinical isolates that were more or less dependent on either Psl or Pel for their biofilm phenotype were nearly entirely dependent on CdrA for biofilm formation. This indicated that, despite the differences in EPS secreted under different conditions or in different strain backgrounds, basic mechanisms for biofilm formation remained consistent across isolates [113,117]. Histological analysis of sputum from CF patients revealed the presence of both Psl and Pel, leading to the formation of large cell aggregates. These aggregates, in combination with the integration of eDNA via binding to Pel, increased the antimicrobial tolerance of biofilms in the host [111]. Indeed, biofilm formation is observed in approximately 80% of all *P. aeruginosa* infections in patients suffering from CF and is the most common trait between different isolates in different niches.

The ability to form biofilms is lost in *P. aeruginosa* laboratory strains that are deficient in QS and, in turn, these strains are typically more susceptible to broad-spectrum antibiotic treatment. As noted in Section 3.2, QS signaling dynamics are complex and highly adaptable. This is relevant when considering that an infection is not a static, single-phase process and the adaptability of *P. aeruginosa* signaling cascades are meant to adjust for a specific environment. For example, a *rhlR* mutant formed a hyper-rugose phenotype when grown on solid media, indicating that it might be pathogenic in a murine infection model [53]. However, the opposite was true, and strains lacking the ability to signal through RhlR were discovered to be non-pathogenic. This is likely due to the fact that RhlR-dependent factors that are required for colonization are absent in the model, negating any deleterious effects that might be associated with the hyper-biofilm formation during the latter stages of infection. Indeed, RhlR-dependent signaling is almost always intact in CF clinical isolates, despite the disruption of the *las* system via inactivation mutations in *lasR*. These findings underscored that infection progression is highly dynamic, with multiple factors feeding into multiple physiological traits that can lead to and sustain a chronic infection. How this occurs remains the focus of ongoing research, especially in the field of CF infection progression.

#### 3.5.4. Hydrogen Cyanide

Much like the other secreted products discussed in this section, hydrogen cyanide production is the result of the convergence of multiple signaling systems. There exist at least two DNA binding motifs consistent with regulation by RhlR and LasR as well as one binding motif for each of the regulators Anr and AlgR/T (Figure 1e) [120]. *P. aeruginosa* is often found in anoxic microenvironments. Anr is an anaerobic transcriptional regulator of arginine deiminase and nitrate reductase, and *anr* is required for the optimal expression of hydrogen cyanide under anaerobic conditions [120]. Conversely, the regulation by AlgR/T is optimal under microaerobic conditions. The mapping of the AlgR/T binding site revealed three distinct transcriptional start sites in the *hcnA* promoter [121]. Ultimately, however, *hcnA* is expressed at a high cell density and is regulated by the *rhl* system. Interestingly, *hcnA* is regulated by RhlR in a RhlI-independent manner as the deletion of *rhlI* had little to no effect on *hcnA* transcript levels [53,83]. However, transcriptome analyses of the RhlR regulon showed that RhlR required the presence of PqsE for optimal expression, indicating that there might be an alternative mechanism of activation for RhlR, beyond the RhlI-synthesized C_4_HSL [82,83]. The regulation of hydrogen cyanide production was determined to be important for pathogenesis. Indeed, hydrogen cyanide was deemed to be a suitable biomarker for a *P. aeruginosa* infection. CF patients with an ongoing *P. aeruginosa* infection produced detectable levels of hydrogen cyanide in both mouth-exhaled and nose-exhaled breaths as measured by mass spectrometry, which indicated that *P. aeruginosa* was producing detectable quantities of hydrogen cyanide in the lower respiratory tract [122,123,124].

In addition to its role as a cytotoxic factor during infection, recent work showed that hydrogen cyanide has an important role in shaping the microbial community, which is particularly relevant considering the intra-clonal diversity that exists during an infection. This is discussed below in Section 3.6. As noted throughout the review, *lasR* mutants are a frequent occurrence in some subpopulations during chronic infection. The loss of *lasR* function could result in the proliferation of “cheater” populations that benefit from the production of secreted products without the metabolic cost of producing them. However, *P. aeruginosa* can police this behavior through the production of hydrogen cyanide. Hydrogen cyanide inhibits cytochrome C oxidase leading to an increase in the levels of ROS. Protection against hydrogen cyanide occurs through the cyanide insensitive terminal oxidase, CioA, and the cyanide sulfur-transferase, RhdA [125]. These genes are regulated by QS only in the presence of cyanide. The absence of QS regulation in a strain lacking *lasR* led to a loss of protection due to a concomitant decrease in the expression of CioA and RhdA. In co-culture experiments, a strain lacking *cioA*, which could still produce hydrogen cyanide, could no longer control the population of a strain lacking *lasR*. These data indicated that CioA was important for self-protection and policing under conditions where “cheater” populations might prosper [125]. Thus, it is critically important for the bacteria to maintain QS signaling and, due to the presence of *lasR* mutant cheater populations, this is likely maintained through the PqsE-RhlR-dependent signaling pathway.

### 3.6. Microevolution of P. aeruginosa in Pulmonary Infections

The long-term survival of *P. aeruginosa* in the host lung is thought to be the result of high levels of intra-clonal diversity induced by mutations, coinciding with the transition from an acute infection to a chronic infection. A single study conducted on samples taken from one CF patient revealed that there were high levels of intra-isolate diversity among the 44 isolates that were obtained [126]. Morphologically similar isolates from one sample revealed highly divergent genotypes, with alterations in several pathways related to growth, virulence factor production, and QS. Interestingly, analysis of a subset of these isolates revealed that recombination, and not random mutagenesis, was the main driver of the genetic diversity in the sample [126]. The genetic diversity of different subpopulations within a single patient was beneficial to the collective; isolates were more susceptible to antibiotic treatment when growing in isolation and resistance to amikacin, ceftazadime, ciprofloxacin, meropenem, tazocin, colistin, tobramycin, and aztreonam increased when samples were grown together [126]. Subsequent studies have used in vitro evolution to assess how clinical isolates can be influenced by members of the resident microbiome [127]. Similar mutations occurred in QS pathways, typically in *lasR* and *pqsR* in the absence or presence of the synthetic CF lung microbiome, indicating that the evolutionary trajectory of *P. aeruginosa* CF isolates was independent of the microbial environment in which they are surrounded [127]. Previous research on this topic indicated that it was much more likely that the environmental pressures within the host lung are what drove the microbial genetic adaption observed in many patients. While the host microbiome does not appear to influence the pathogenicity of *P. aeruginosa*, the pathogen can affect the microbial diversity of a patient. Patients infected with an epidemic strain of *P. aeruginosa* had lower microbial diversity and enrichment of other lung pathogens, such as *Streptococcus* spp., indicating that enhanced pathogenesis of certain strains can be the result of not only the infecting strain of *P. aeruginosa*, but might also be related to pathogen-pathogen interactions in the lung [128].

Furthermore, there appears to be variation in the actual diversity. Isolates that overproduce secreted virulence factors can co-exist with populations that do not produce secreted virulence factors [129,130]. Where these two distinct subpopulations arise from is still unknown. However, it is possible that two distinct infections can occur simultaneously in which one subpopulation exhibits characteristics associated with chronic infection while the other exhibits characteristics associated with acute infection. A study of four clinical isolates showed a time-dependent production of elastase, pyocyanin, and pyoverdine. Strains from naïve, acute, and chronic infections lasting less than six months produced anywhere between 2- and 6-fold more of these factors compared to isolates from chronic infections lasting more than 6 months [131]. Curiously, while levels of the examined secreted virulence factors decreased concomitantly with 3OC_12_HSL and PQS levels, C_4_HSL levels remained steady across isolates, indicating a *rhl* independence that might be critical to the maintenance of chronic infections [131]. Indeed, a longitudinal study of several clinical isolates from different sources of infected hosts revealed that LasR-independent transcription and biofilm growth and maintenance were reliant on RhlR-dependent transcriptional activity [132]. Interestingly, *rhlR* regulation in *lasR* mutant strains was shown to proceed via PhoB in phosphate limiting conditions in laboratory strains, but this requirement was not observed in clinical isolates, indicating that there are multiple factors responsible for the maintenance of RhlR-dependent signaling in these isolates (Figure 1d) [81].

A recent study of CF patients from Denmark revealed that half of the participants in the study that had an “eradicated” *P. aeruginosa* infection were, in fact, not cleared of the infection [133]. In most cases, these individuals were re-infected by the same strain that was previously found to reside in their lungs, indicating that they were being re-infected not from the environment, but from a persisting reservoir within the body [18]. This was consistent with other case reports that identified the paranasal cavity as a reservoir for *P. aeruginosa* that can promote re-infection. Thus, not only can *P. aeruginosa* tailor its virulence signaling cascade to adapt to its environment, but it can also persist and prime its pathogenesis for subsequent re-infections, presumably throughout the course of a patient’s life.

### 3.7. The Role of QS in UTIs and Bound Wounds

The type of infection and the environment in which it exists influence the signaling cascades that induce virulence. While several burn models have been established in porcine, rodent, and even invertebrate systems, there are likely key differences in the host’s response that alter signaling. One such study that attempted to overcome this issue used human burn wound exudate (BWE) to assess transcription in the laboratory strain PAO1 [134]. As expected, QS was upregulated, as well as pathways required for iron scavenging. However, unexpectedly, the pattern of QS activation was different when compared to standard laboratory growth conditions. In standard media, QS signaling progressed canonically and remained high at an OD_600 nm_ = 4.6. However, in BWE, QS progressed differently in two important ways in BWE: (i) it was induced earlier than control strains under standard laboratory growth conditions, especially with respect to the PQS pathway, and (ii) at OD_600 nm_ = 4.6, where expression was the highest in standard media, expression of all QS regulators dropped by approximately 50% except for *rhlr*, which remained expressed to levels of earlier timepoints. Consistent with the notion that rhlr is the main driver of qs-dependent virulence in acute and chronic infections, *lasb* expression remained high even after the decline in *lasr* expression in BWE [134].

The mechanism of disease progression and its reliance on QS is less clear in uti models. It has been noted that the *rhl* system is required for pathogenesis; deletion of *rhli* in a murine UTI model led to reduced virulence, which could only be recovered by the addition of c_4_hsl [135]. Histological data supported these findings with fewer bacteria detected in the bladder and kidneys of mice exposed to QS deficient strains compared to WT. Additionally, QS mutants induced a lower inflammatory response compared to the WT strains in a murine infection model. Indeed, the presence of an intact QS signaling system appears to be a hallmark of clinical isolates obtained from patients suffering from acute UTI. However, variability of QS-dependent gene expression was observed among clinical isolates. As we noted in Section 3.2, QS-dependent gene expression can be affected by the host; clinical isolates obtained from patients with a UTI exhibited reduced pyocyanin, elastase, and rhamnolipid production when exposed to urine. It is within this model of chronic UTI that EPS-independent biofilms formed, circumventing the requirement of QS for infection [24]. It was postulated that this is likely the case with most types of chronic infections. QS is required for the acute infection and the transition to chronic infection, but once at that stage, QS could become dispensable. However, it is unclear what social advantage these QS deficient strains might have if they are expelled back into the environment. It is important to remember that while infections are conceptualized as being caused by a homogenous strain, there are microenvironments within the host that will allow one population to behave differently from another genotypically identical population elsewhere in the host.

The propensity for *lasR* to accumulate mutations indicates that there is likely a *lasR*-dependent exoproduct that is either (i) no longer required for infection or (ii) energetically expensive, which would result in a decidedly important growth advantage for strains not expressing it [79]. Irrespective of the benefits this type of evolution might have for *P. aeruginosa*, the ability to bypass *lasR* and activate RhlR points to the balance between the growth advantages of cheating off public goods (*lasR* mutants) and the progression of virulence mediated by activation of RhlR. Indeed, targeting PqsE, RhlR, and RhlI via small-molecule inhibition is an attractive model for the development of new antimicrobial therapies. The promise of disrupting the *rhl* system as well as other potential therapies will be discussed in Section 5.1.

## 4. Intrinsic Antibiotic Resistance Mechanisms

### 4.1. Biofilms

Biofilm formation has long been studied as a mechanism of antibiotic tolerance. As discussed in Section 2.2.2, *P. aeruginosa* colonizes both medical equipment and CF patient lungs by forming biofilms, with CF patients experiencing early childhood colonization that develops into chronic, life-long infections. A study comparing biofilm formation and antibiotic resistance of CF patients and those with VAP revealed that isolates from ventilated patients displayed robust and consistent biofilm formation and higher antibiotic resistance than CF isolates [136]. For almost all isolates, however, the biofilm inhibitory concentration for drugs tested was higher than liquid culture minimal inhibitory concentration (MIC) suggesting that biofilms are a barrier to antibiotic efficacy regardless of the infection type.

Biofilms confer resistance to numerous antibiotics. A study of nearly forty multidrug-resistant *P. aeruginosa* isolates in Nigeria identified 50% resistance to gentamicin, and approximately 30% resistance to imipenem, aztreonam, and cefepime [137]. The cohort was unanimously resistant to other cephalosporins, penicillin, ciprofloxacin, and nitrofurantoin. Resistance to quinolones and gentamicin was consistent regardless of individual biofilm-forming capability, while strong biofilm formers were more resistant to imipenem and aztreonam. Other studies have corroborated the link between biofilm formation and carbapenem resistance. One such study showed that the majority (88%) of carbapenem-resistant *P. aeruginosa* isolates from a hospital in Korea formed robust biofilms and were also resistant to amikacin, ceftazidime, and cefepime [138]. Importantly, only 19% of isolates harbored an acquired plasmid-borne carbapenemase gene (either *bla*_IMP_ or *bla*_VIM_) suggesting that biofilm formation was the main driver of antibiotic resistance in these isolates.

Antibiotic misuse is dangerous because the treatment of infections with sub-inhibitory concentrations can evolve biofilm-conferred resistance. Generationally cultured *P. aeruginosa* biofilms in sub-MIC concentrations of ciprofloxacin evolved increased resistance. Planktonic cultures also evolved higher ciprofloxacin MICs, although more passages were required to induce resistance than for biofilm populations [139]. Importantly, biofilms passaged without ciprofloxacin also evolved higher resistance, demonstrating that premature cessation of antibiotic regimens can allow persistent populations to re-grow and form biofilms, thus resistance to the drug and ultimately rendering it ineffective.

As biofilm formation is regulated by QS, there has been research on whether quorum quenching could sensitize *P. aeruginosa* to antibiotics. Disruption of biofilm formation through perturbation of PQS-dependent QS sensitized *P. aeruginosa* to tobramycin and resulted in decreased virulence factor production [140]. An essential component of the biofilm matrix is eDNA. Secretion of eDNA is also mediated by QS; *lasR*, *rhlR,* and *pqsA* mutants produce lower levels of eDNA and, consequently, form weaker biofilms compared to WT *P. aeruginosa* [141]. When exposed to tobramycin in vitro, QS mutant biofilms were killed after 24 h, whereas mutant biofilms supplemented with eDNA remained tolerant. Furthermore, the addition of eDNA increased the bactericidal MICs of tobramycin and gentamicin for WT and mutant biofilms, demonstrating that eDNA protects against aminoglycosides [142]. In addition to the interaction between eDNA and antibiotics, phenazines and cellular metabolism play a critical role in conferring tolerance to antibiotics. A recent study showed that biofilms grown in the absence of phenazines were more susceptible to ciprofloxacin treatment. Interestingly, this benefit was independent of efflux pump systems or biofilm matrix components. Phenazine production led to a distinct subpopulation of cells in the hypoxic region of the biofilm that expands the metabolic versatility of the biofilm, indicating that metabolism can directly affect tolerance to antibiotics [115].

### 4.2. Outer Membrane Porins

*P. aeruginosa* is intrinsically resistant to antimicrobials in part because of its impermeable outer membrane (OM). Unlike *E. coli*, *P. aeruginosa* does not possess freely diffusible pores and instead utilizes substrate-selective porin channels. For the scope of this review, the focus is on porins related to antibiotic resistance and pathogenicity. The peptidoglycan-associated lipoprotein (PAL) OprF, homologous to OmpA from *E. coli*, is rarely completely open, with its default state being closed or weakly permissible [143]. OprF is a structural porin, contributing to OM integrity and stability by forming complexes with other integral membrane proteins. OprF is a constituent of bacterial outer membrane vesicles (OMVs), which are endocytic blebs secreted by Gram-negative bacteria that confer virulence and stress-response functions. Alternative to its role in QS signaling through PqsR binding, PQS interacts with lipopolysaccharides (LPS) in the OM to induce OMV budding [144]. OprF expression is linked to OMV secretion, as an *oprF* mutant demonstrated an 8-fold increase in OMV secretion compared to WT [145]. Another study showed OMV shedding along the phagolysosome membrane of *P. aeruginosa* infected macrophages in both WT and *oprF* strains [146]. Moreover, an *oprF* mutant showed increased PQS production and secretion of OMVs [145], suggesting OMV budding induced by OprF is PQS-dependent. Indeed, *oprF* deletion resulted in increased HHQ and delayed production of PQS [147]. Therefore, induction of OMV budding via the loss of *oprF* might occur through modulation of QS. OMVs are thought to confer resistance to colistin and polymyxin-B through drug adsorption. Thus, OprF-mediated induction of OMV blebbing could contribute to *P. aeruginosa* antibiotic resistance [148].

OprF is an abundant constituent of biofilm secretions, yet information on its role in biofilm formation is inconsistent. Biofilm formation is promoted through diguanylate cyclase synthesis of c-di-GMP, inducing the shift in *P. aeruginosa* from a planktonic to a sessile state. Metabolically dormant bacteria are preserved in the biofilm center, facilitating antibiotic resistance and bacterial persistence. An *oprF* mutant that has increased c-di-GMP levels forms robust biofilms and overexpresses EPS in aerobic conditions [149]. However, *oprF* mutants, like QS deficient mutants, form poor biofilms in anaerobic conditions [150]. OprF was found to be upregulated in acute and chronic murine infection models, and transposon mutagenesis revealed no insertions in OprF [151], indicating its indispensable role in *P. aeruginosa* infection. *P. aeruginosa* forms biofilms in the airway of CF patients, which is an anaerobic niche due to thickened mucus. Therefore, OprF might indirectly impact the biofilm formation of *P. aeruginosa* in a CF airway infection niche via QS modulation. Indeed, OprF is upregulated under anaerobic conditions and secreted in CF patient lungs [152], indicating another potential role for OprF in *P. aeruginosa* pathogenesis.

Consistent with evidence that OprF is implicit in biofilm formation and QS modulation, *oprF* deletion attenuated *P. aeruginosa* virulence. An *oprF* mutant showed impaired type III secretion system (T3SS) activity and expression, the delivery system of several *P. aeruginosa* virulence factors. T3SS expression is inversely correlated to c-di-GMP levels. *oprF* deletion resulted in increased c-di-GMP levels relative to WT, and decreased T3SS expression, suggesting that OprF is a positive regulator of T3SS [153]. Exotoxin effectors ExoS and ExoT are not secreted in an *oprF* mutant, yet are also not accumulated in the cytosol, suggesting that OprF expression is also linked to the transcription of these virulence genes. Indeed, pyocyanin production decreased in an *oprF* deficient strain, while *lasB* expression was delayed but not abrogated [147]. The SigX (a σ-factor) regulon includes porins *oprF* and *oprD* (discussed below) and QS controlled virulence genes (phenazines, hydrogen cyanide, type IV pili, c-di-GMP metabolism, and exotoxin), supporting the link between *oprF* expression and virulence phenotypes. In one study, *oprF* mutants were more resistant to piperacillin and more susceptible to tetracycline [154]. However, the contribution of OprF to antibiotic resistance is unlikely to be a direct effect of the porin function itself and probably an indirect role through the QS-regulated mechanisms described here.

Another porin for which altered expression can directly confer antibiotic resistance is OprD, a channel for amino acid uptake. Carbapenems, like imipenem and meropenem, bind inside the OprD channel. These drugs were rendered ineffective when *oprD* expression levels were decreased or when the OprD channel was altered structurally [155,156,157]. Meropenem hypersusceptibility is conferred by a divergence of a ten amino acid sequence divergence in the C-terminal L7 loop of OprD, but imipenem resistance profiles are unaltered [156]. Heteroresistance to imipenem, but not meropenem, was observed in some *P. aeruginosa* strains lacking OprD due in part to overexpression of a homologous porin channel, OpdP [158]. Differential expression of these homologous porins is carbon-driven and coordinated by RNA regulatory molecules CzcR and Hfq [159]. Importantly, carbapenem resistance is conferred through a synergistic combination of mechanisms, including outer membrane porins, efflux pumps (discussed below), chromosomal and plasmid-borne carbapenemases, and those yet to be identified.

Negative regulation of *oprD* occurs through two functionally redundant regulatory systems: CopR-CopS and CzcR-CzcS. Upon metal binding of the histidine kinase (CopS or CzcS), the response regulator (CopR or CzcR) is phosphorylated and actively represses *oprD* transcription [160]. Hfq is required for CzcR localization to the *oprD* promoter and CopR-mediated *oprD* repression [161]. In addition to downregulating *oprD*, CzcR regulates *P. aeruginosa* QS and virulence genes. A *czcRS* mutant showed increased pyocyanin production through transcriptional de-repression of the *phzA1* promoter [161]. Likewise, a *czcRS* mutant also showed downregulation of *rhl*, *lasB*, and *pqsH* [161], impaired biofilm formation, and expression of rhamnolipids [162]. AI concentrations were also reduced. Altogether, this suggests CzcR is a negative regulator of pyocyanin production, but a positive regulator of QS and biofilm formation. Indeed, CzcR binds the *lasI* promoter [161], suggesting that it might antagonistically compete with the negative QS regulator RsaL (Figure 1d). The CzcSR system might therefore serve as an attenuator of *P. aeruginosa* virulence and antibiotic resistance based on metal bioavailability. Indeed, iron uptake is important for driving bacterial competition and soliciting virulence phenotypes related to nutrient scavenging during infection. Furthermore, because OprD and QS genes share a common regulator, it is possible that OprD is involved in a QS-mediated virulence pathway.

### 4.3. Efflux Pumps

**Figure 2:**
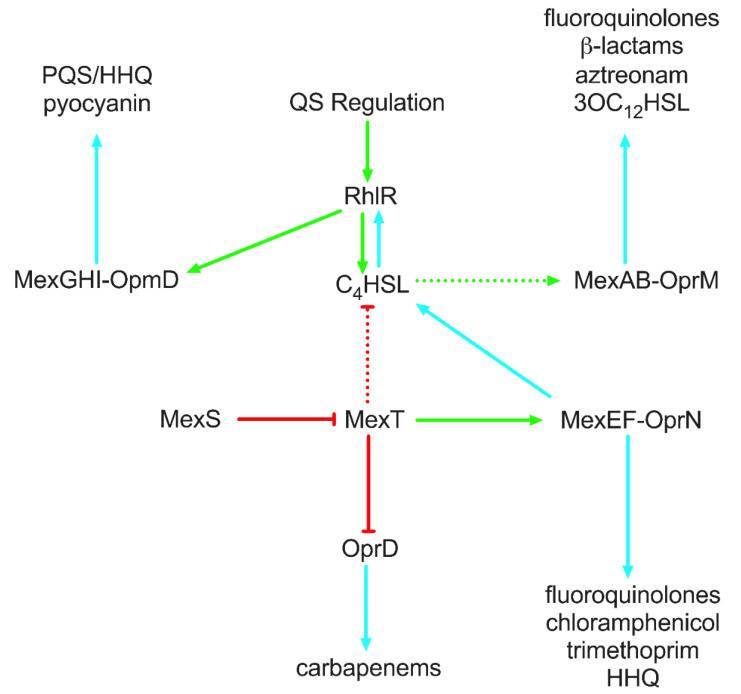
The interplay between QS and intrinsic mechanisms of antibiotic resistance. Select regulators of efflux pump and porin function as they relate to QS regulation. RhlR binds to C_4_HSL (blue arrow) and activates the transcription of the synthase responsible for C_4_HSL (green arrow). RhlR and C_4_HSL directly regulate the expression of *mexGHI*-*opmD* (green arrow) and indirectly regulates *mexAB-oprM* expression via an unknown mechanism. MexGHI-OpmD plays a role in secreting important virulence factors (blue arrow). MexAB-OprM expels fluoroquinolones, β-lactams, aztreonam, and AI signaling from the cell. MexT indirectly represses (dotted red bar) the production of C_4_HSL, while simultaneously upregulating the expression of *mexEF-oprN*. MexEF-OprN expels C_4_HSL, fluoroquinolones, chloramphenicol, trimethoprim, and HHQ from the cell (blue arrows). MexT also represses *oprD* expression, which contains a carbapenem binding pocket (blue arrow).

Several resistance-nodulation-cell division (RND) efflux systems are known to confer antibiotic resistance in Gram-negative bacteria. Regulation of these efflux systems is intricate and an area of ongoing research. The MexAB-OprM efflux system is the most well-studied in the context of *P. aeruginosa* antibiotic resistance. Transcriptional analyses of *mexR* (*nalB*) mutants led to an overexpression of the *mexAB-oprM* operon, resulting in increased MICs to many currently used antibiotics, including last-line drugs like fluoroquinolones, β-lactams, and aztreonam [163,164,165]. Likewise, CpxR upregulation of the MexAB-OprM efflux system in *mexR* laboratory and clinical strains increased the MICs for ciprofloxacin, ofloxacin, ceftazidime, cefsulodin, and aztreonam [166]. *nalC* and *nalD* mutants also conferred increased antibiotic resistance but to a lesser degree than *mexR* mutants. In addition to drug efflux, MexAB-OprM extrudes 3OC_12_HSL (Figure 2) [167]. The expression of MexAB-OprM is dependent on high cell density, but more specifically the concentration of C_4_HSL [168]. Although the mechanism is unknown, it was suggested that C_4_HSL either directly induced expression of the MexAB efflux system or interfered with MexR repression of the system. Furthermore, it was proposed that C_4_HSL concentration has a direct role in regulating the inverse relationship between MexAB-OprM and MexEF-OprN expression (Figure 2) [169].

MexEF-OprN effluxes chloramphenicol, fluoroquinolones [170], and trimethoprim (Figure 2) [171]. Mutations in the *nfxC* gene and/or constitutive expression of MexT through alteration of residue G257 caused the overexpression of the MexEF-OprN efflux system [171]. Expression of the MexEF-OprN and MexAB-OprM systems is mutually exclusive, as MexT represses the MexAB-OprM system and *nfxC* mutants downregulate *mexA* and *oprM* genes. It was suggested that MexEF-OprN disrupts C_4_HSL activation of MexAB-OprM, although the exact mechanism is unknown. Possibilities include: (i) transcriptional repression of the C_4_HSL synthase gene *rhlI* or (ii) MexEF-OprN-mediated extrusion of C_4_HSL. The former explanation is supported by evidence that MexT antagonizes C_4_HSL production while promoting MexEF-OprN expression [168]. MexT is also a repressor of the OprD porin (discussed above) and its constitutive expression in clinical isolates increases resistance to carbapenems (Figure 2). MexS is the cognate repressor of MexEF-OprN and mutations in *mexS* confer multidrug resistance [170,172], presumably through upregulation of the MexAB-OprM system. *mexST* mutants overexpressing MexEF-OprN demonstrated impaired pyocyanin production suggesting that (i) MexEF-OprN repression is dominant and (ii) MexEF-OprN could be involved in the secretion of pyocyanin [171]. Indeed, MexEF-OprN effluxes HHQ and kynurenine, precursors of PQS [170]. MexEF-OprN expression is also inversely correlated to biofilm formation. Altogether, the evidence suggests that MexAB-OprM efflux and QS are regulated in parallel, whereas MexEF-OprN efflux is oppositely regulated.

QS mediates the secretion of proteases required for bacterial growth on limited nutrient substrates. As discussed in Section 3.3 clinical isolates naturally evolve *lasR* mutations during chronic infection, forming “cheater” populations that effectively cease protease secretion in favor of utilizing public goods, thereby establishing a fitness advantage [173,174]. *lasR* strains grown on casein evolved an inactivating mutation in *mexT*, resulting in downregulation of the *mexEF-oprN* efflux system and increased susceptibility to chloramphenicol [175]. A similar phenomenon has been described in *Chromobacterium violaceum,* which possesses the LuxR/I QS homologs CviR/I. Spontaneous cheater mutants derived from a *cviR* mutant strain were more susceptible to bactobolin and tetracycline [176]. Therefore, QS regulation of efflux pump expression in Gram-negative bacteria directly affects antibiotic resistance.

Like the MexEF-OprN efflux system, the MexCD-OprJ system was implicated in QS AI extrusion. MexCD-OprJ is repressed by NfxB, and *nfxB* mutant clinical isolates overexpress MexCD-OprJ [177]. Overexpression of MexEF-OprN and MexCD-OprJ is correlated to deficient production of QS-regulated virulence factors. A *mexD* deletion in a *nfxB* mutant restored QS mediated virulence, demonstrating that QS disruption in a MexCD-OprJ overexpressing strain is NfxB-independent [178]. Analysis of PQS:HHQ ratio levels in *nfxBmexD* mutant supernatants and cell lysates revealed deficient HHQ secretion in *nfxBmexD* mutant strains, suggesting that MexCD-OprJ mediated extrusion of HHQ disrupted the PQS system signaling. Moreover, the expression of *pqsE* decreased in strains overexpressing *mexCD-oprJ*, while HSL AI secretion was unaffected [178]. Therefore, the MexCD-OprJ efflux system is exclusively linked to the secretion of effector molecules produced by the PQS QS circuit.

Another efflux system that is implicated in PQS signaling is MexGHI-OpmD. This efflux system is unusual in that it possesses a fourth subunit of unknown function, MexG, a DoxX family protein. *mexG* was downregulated in a *pqsR* deletion strain, suggesting it is a target gene in the PqsR regulon [179]. Affinity probe assays revealed MexG binds soluble PQS [180]. Likewise, whole-cell lysates from a *pqsB* mutant unable to produce PQS failed to capture MexG, confirming that PQS binds MexG. Co-immunoprecipitation showed that MexG also binds the PQS precursor molecule HHQ. Mutagenesis of the MexG DoxX domain, a transgenic loop region, disrupted PQS binding [180]. These data suggest a potential role for MexG as a receptor for PQS and that PqsR and MexG might compete for PQS binding. Alternatively, PQS/HHQ could be a substrate of the MexGHI-OpmD efflux pump [181]. Indeed, our own data showed that mutations that disrupted the PqsE-RhlR interaction lead to the downregulation of *mexG* and *opmD* genes, suggesting MexGHI-OpmD is controlled by QS signaling [83]. Furthermore, MexGHI-OpmD has been implicated in the secretion of pyocyanin, linking the expression of the phenazine operon with that of its secretion apparatus [181].

## 5. Future Prospects & Conclusions

### 5.1. Quorum Quenching in P. aeruginosa: The PqsE-RhlR Interaction Represents Renewed Hope for a Viable Anti-QS Therapeutic

The idea of attenuating *P. aeruginosa* virulence through the manipulation of QS is not new [68,84,182,183,184,185,186]. It is an attractive alternative to traditional antibiotics because (i) QS is not required for growth and should not lead to the development of mechanisms of resistance and (ii) QS is thought to be the norm in the bacterial world with a few exceptions, making it generally applicable to multiple pathogenic systems. However, success in this realm has been incremental and, to the best of our knowledge, no molecule has reached the level of clinical trials. Here, we propose that targeting the PqsE-RhlR interface is a novel, viable option for drug development because of its unique mechanism of action. Additionally, other areas of ongoing research that could lead to the development of a viable treatment alternative to traditional antibiotics for *P. aeruginosa* infections are highlighted.

Sitting atop the canonical QS hierarchy, LasR was deemed to be the primary target for drug discovery for many years [187,188,189]. Indeed, it possessed some ideal characteristics: (i) it has a ligand-binding pocket from which to start a medicinal chemistry approach, and (ii) inhibiting the master QS regulator required for virulence factor production should prevent infection. The latter has been assessed throughout this review. The requirement for LasR during infection can be bypassed; inactivating mutations in *lasR* would render any treatment targeting LasR function ineffective. Regarding the former, the LasR binding pocket was shown to be remarkably plastic in its ability to bind a diverse set of molecules, both AI derivatives and synthetic small molecules, allowing it to remain functional as an activator in *E. coli* reporter systems and in *P. aeruginosa* [189,190]. This was somewhat surprising given that many of the synthetic small molecules analyzed were derivatives of the small molecule inhibitor chlorolactone (CL), which was shown to inhibit CviR function [191]. CviR is a LuxR-type protein from *C. violaceum*. Structural analysis of CviR bound to CL revealed a closed conformation of the DBD, resulting in a fold that could no longer bind DNA. However, CL and its derivative meta-bromothiolactone (mBTL), were found to be activators of both LasR and RhlR, indicating that a one size fits all drug targeting scheme aimed at inhibiting LuxR-type receptors to suppress virulence is ill-conceived [67,189]. We propose that the best targets for anti-QS therapeutics are PqsE and RhlR by means of allosteric inhibition of both targets.

**Figure 3:**
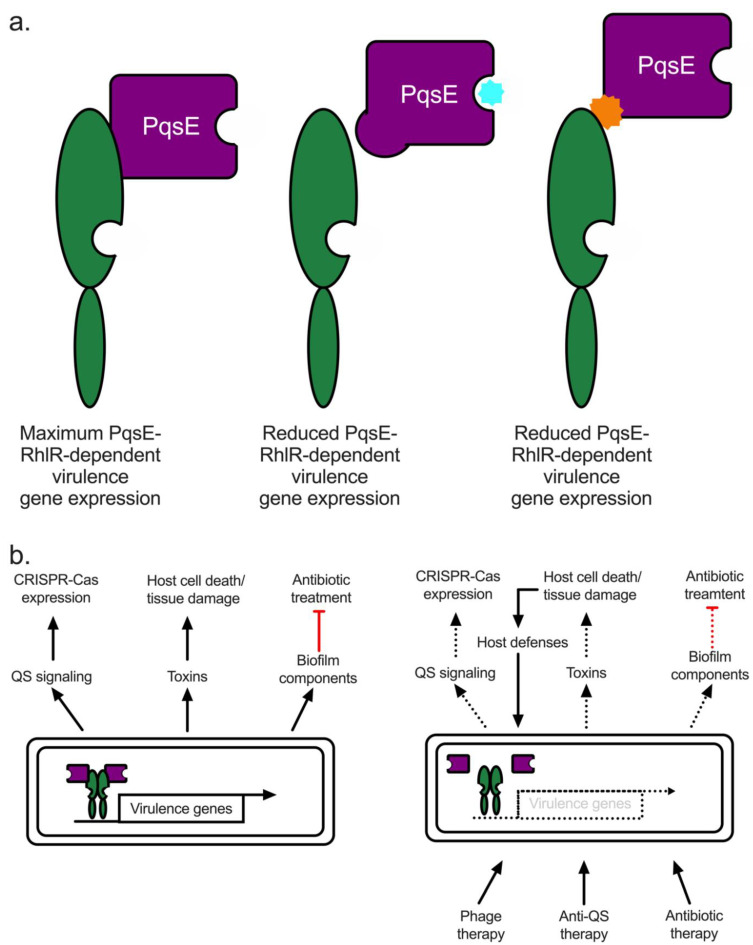
The future of anti-infective therapeutics to treat *P. aeruginosa* infections. (**a**) Schematic representation of how disrupting the PqsE-RhlR interaction could affect RhlR-dependent transcription. An inhibitor that binds in the catalytic pocket could disrupt the PqsE-RhlR interface by allostery (middle) or via a direct perturbation of the buried surface area between PqsE and RhlR (right). (**b**) Flow chart detailing how combinatorial treatment with phage therapy, anti-QS therapy, and antibiotic therapy could enhance the efficacy of each individual therapy, creating a new viable option for the treatment of antibiotic-resistant bacteria.

While the PqsE catalytic pocket was probed by medicinal chemists with multiple molecules that successfully inhibited PqsE catalytic function, the molecules were ineffective at suppressing virulence [84]. This was consistent with our recent work indicating that the main role for PqsE in virulence is through binding to RhlR and the regulation of RhlR DNA binding, not through its catalytic function within the PQS biosynthetic pathway. Interestingly, we showed that the PqsE-RhlR interaction can be disrupted via mutagenesis in two ways. First, the interaction was disrupted by the large, bulky side-chain substitutions in residues that bind to inhibitor molecules. Indeed, these mutations were designed to mimic the inhibitor-bound state of PqsE. Second, we probed the surface of PqsE for positively or negatively charged residues that could represent the interaction interface between PqsE and RhlR and discovered three arginine residues that were key to complex formation. Thus, based on our work, we propose that there are two distinct mechanisms of targeting RhlR function through the PqsE-RhlR complex without targeting the ligand-binding pocket of RhlR: (i) binding to the catalytic pocket of PqsE, resulting in a structural change in the interface between PqsE and RhlR that disrupts the interaction and thus, RhlR function, and (ii) small molecule binding directly to the interface between PqsE and RhlR, resulting in a loss of complex formation and, similarly to (i), RhlR function, effectively abrogating virulence and pathogenesis (Figure 3a). The mechanism by which targeting the catalytic pocket disrupts the PqsE-RhlR interaction is the focus of ongoing structural work.

### 5.2. Alternative Therapeutics: Phage and Antibody Therapies

Phage and antibody therapies have been applied to other systems. Here, a handful of recent publications that highlight the utility of these potential therapies in a variety of disease models will be reviewed.

Phage therapy has been explored as a viable therapeutic against bacterial infections for over a century. Logically, this makes sense: bacteriophages exist to infect and proliferate using a bacterial host. However, phage therapy relies on extensive knowledge of phage biology and reproductive cycle. There are more bacteriophages on Earth than any other “organism” and they are just as diverse in their mechanisms of replication and reproduction as anything encountered in biology. Recent studies have used phage therapy in a variety of animal models such as *K. pneumoniae* in pulmonary infections and even *P. aeruginosa* in skin infections, as well as other infections using invertebrate and murine models. Interestingly, a CF model with zebrafish embryos was recently developed. This model established the usefulness of phage therapy combined with traditional antibiotic treatment. CF zebrafish embryos infected with the laboratory strain PAO1 experienced approximately 80% lethality [192]. This level of lethality was cut in half when the embryos were treated using a phage previously known to kill *P. aeruginosa* in vitro. Ciprofloxacin had nearly the same effect on reducing the percent lethality. When combined, the therapies exhibited a somewhat synergistic effect as the overall percent lethality dropped to approximately 30% [192]. Phage therapy has been performed in a *P. aeruginosa* burn wound model and showed similarly positive effects to the CF zebrafish model [193]. In recent years, the efficacy of phage therapy has proven beneficial in a small collection of clinically relevant environments, such as in a 26-year-old CF patient, lung transplant patients, and patients with *S. aureus* chronic sepsis [192,194,195,196]. Importantly, in some bacterial systems, such as the laboratory strain PA14 *P. aeruginosa*, the bacterial immune response (CRISPR-Cas systems) that can detect and eliminate phage infection is controlled by QS [197,198]. Thus, repressing QS could have the secondary benefit of making phage therapy more potent. It is potentially exciting to envision a multi-therapy approach whereby anti-QS, phage, and antibiotic therapies are administered to reduce the reliance on any one therapy and to potentially enhance the effectiveness of all treatments in a synergistic manner (Figure 3b).

While anti-QS, antibiotic, and phage therapy require an outside “force” to help clear infection, antibody therapies take advantage of our natural defenses to elicit an appropriate response in the face of a life-threatening bacterial infection. As discussed in Section 4.2, *P. aeruginosa* produces OMVs, small membrane blebs containing lipids, proteins, and small molecules from the outer membrane of the pathogen. Recent work has been performed to exploit this natural biological process to assist in presenting antigens to the immune system in a much more effective manner than conventional conjugate vaccines. Researchers used a recombinant *E. coli* expression system to produce the cell-surface polysaccharide poly-*N*-acetyl-D-glucosamine (PNAG) from *S. aureus* to yield OMVs containing PNAG [199]. This method provided a way to test whether OMVs could be used as a delivery system for antigen presentation. Mice vaccinated with OMVs containing PNAG produced antibodies specific towards PNAG. The immune response elicited from these antigens provided protective immunity against *S. aureus* in a murine model, as 70% of mice survived compared to 10% in the mock control treatment [199]. Such studies using *P. aeruginosa* factors as potential antigens for the development of antibody therapies are less developed and require further characterization [200].

### 5.3. Conclusions

While this review highlights many of the strides the field has made in the past five years, especially as it relates to virulence in the host, there is still a great deal we do not know about *P. aeruginosa* pathogenesis. This is true for all the ESKAPE pathogens, making research that unites basic and clinical principles paramount to finding viable treatment options for these types of infections. With much of the current research on ESKAPE pathogens focused on acquired mechanisms of antibiotic resistance, we purposefully highlighted many of the gaps in our understanding of intrinsic antibiotic resistance mechanisms throughout the review and we are confident that they can and will be addressed by the field (Box 1). Continued research of *P. aeruginosa* intrinsic resistance mechanisms is crucial for a holistic understanding of pathogenesis and effective treatment of infections.

## Figures and Tables

**Table 1:** Antibiotic resistance trends in clinical isolates.

Antibiotic	Class	Mechanism of Action	Total Resistance (%)	Isolates Tested	Trend	HAI Type with Highest% Resistance
Imipenem, meropenem	Carbapenem	Peptidoglycan crosslinking/cell wall synthesis	13.3%	6665	−1%	CLABSI
Gentamicin, tobramycin, amikacin, streptomycin	Aminoglycoside	Protein synthesis	8.5%	7931	−0.6%	CAUTI
Ciprofloxacin, levofloxacin	Fluoroquinolone	Topoisomerase/DNA synthesis	15.2%	7795	−1.6%	CAUTI
Cefazolin, cefepime, ceftriaxone, ceftazidime	Cephalosporin	Peptidoglycan/cell wall synthesis	15.1%	7879	−0.9%	CLABSI/CAUTI
Piperacillin, tazobactam	Penicillin	Peptidoglycan crosslinking/cell wall synthesis	11.9%	7467	−0.1%	CLABSI/CAUTI
	Multi-drug		7.9%	7940	−2.1%	CAUTI

CLABSI = central line-associated bloodstream infection. CAUTI = catheter-associated urinary tract infection.
